# Supplementary material for: QMAP: a benchmark for standardized evaluation of antimicrobial peptide MIC and hemolytic activity regression
Source: Sci Rep. 2026 Jun 3;16:25387. doi: 10.1038/s41598-026-56004-8 (PMC13473575; doi:10.1038/s41598-026-56004-8)
Supplement: Supplementary file 1 [file 41598_2026_56004_MOESM1_ESM.pdf]

Supplementary Material

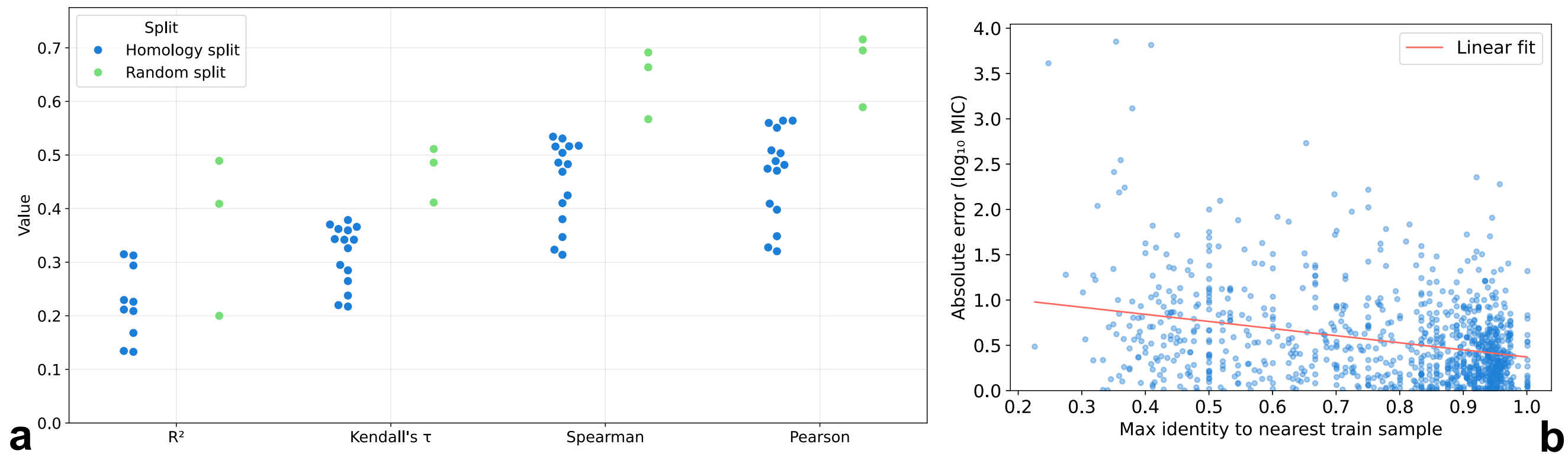

Figure S1. Visualization of potential overfitting when no homology-based split is applied. **a**: Swarm plot of correlation metrics (R^2, Kendall's τ, Spearman, and Pearson) for models trained and evaluated using either a homology split or a random split. For the homology split, the three evaluated models are each assessed across five benchmark sets, yielding 15 individual dots. For the random split, each of the three models is evaluated on a single randomly obtained test set, yielding 3 individual dots. **b**: Scatter plot of the absolute prediction error (log_{10} MIC) as a function of the maximum sequence identity to the nearest training sample, for the linear model trained on the random split. A linear regression fit (red line) reveals a descending trend (Pearson r = -0.31), indicating that predictions deteriorate for samples with low identity to the training set.

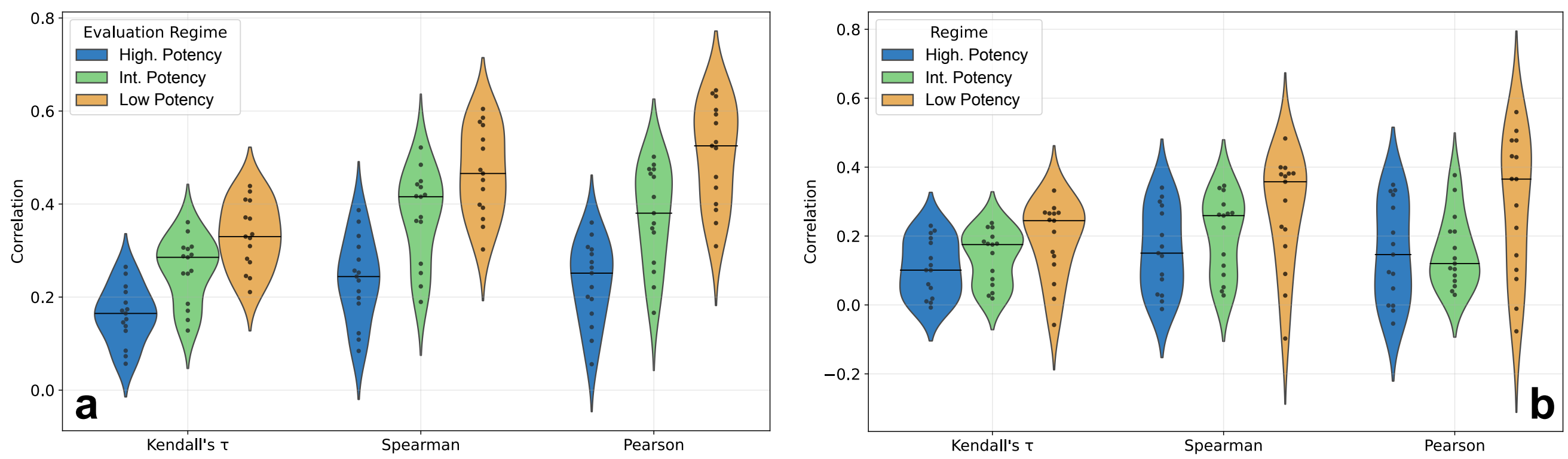

Figure S2. Investigating the high-potency regime collapse. **a:** Violin plots with overlaid swarm plots representing stratified correlation scores (Kendall's τ, Spearman, and Pearson) for all three models evaluated under the homology split, trained on the full dataset. Each dot represents the correlation achieved on the stratified subset of one of the five benchmark test sets, yielding 15 points per violin. The horizontal line denotes the median. Three stratification levels are used: High Potency (MIC $< 10\ \mu$M), Intermediate Potency ($10\ \mu\text{M} \leq \text{MIC} < 100\ \mu$M), and Low Potency (MIC $\geq 100\ \mu$M). A consistent degradation of correlation is observed as peptide potency increases. **b:** Violin plots with overlaid swarm plots representing the same stratified correlation scores, but where both training and evaluation are restricted to the corresponding MIC stratum — that is, each model is retrained on the subset of training data matching the potency threshold. Notably, the High Potency stratum retains the largest training set, followed by Intermediate Potency and Low Potency. Results aggregate all three models across five benchmark splits, yielding 15 points per violin. Despite this data enrichment strategy, correlation performance continues to degrade with increasing peptide potency.
